# Feature Selection for Health Care Costs Prediction Using Weighted Evidential Regression †

**DOI:** 10.3390/s20164392

**Published:** 2020-08-06

**Authors:** Belisario Panay, Nelson Baloian, José A. Pino, Sergio Peñafiel, Horacio Sanson, Nicolas Bersano

**Affiliations:** 1 Department of Computer Science, Universidad de Chile, Santiago 8320000, Chile; jpino@dcc.uchile.cl (J.A.P.); spenafie@dcc.uchile.cl (S.P.); 2 Allm Inc., Tokyo 150-0002, Japan; horacio@allm.inc (H.S.); n.bersano@allm.inc (N.B.)

**Keywords:** health care costs, dempster–shafer theory, supervised learning, regression, feature selection, evidential regression, interpretable prediction

## Abstract

Although many authors have highlighted the importance of predicting people’s health costs to improve healthcare budget management, most of them do not address the frequent need to know the reasons behind this prediction, i.e., knowing the factors that influence this prediction. This knowledge allows avoiding arbitrariness or people’s discrimination. However, many times the black box methods (that is, those that do not allow this analysis, e.g., methods based on deep learning techniques) are more accurate than those that allow an interpretation of the results. For this reason, in this work, we intend to develop a method that can achieve similar returns as those obtained with black box methods for the problem of predicting health costs, but at the same time it allows the interpretation of the results. This interpretable regression method is based on the Dempster-Shafer theory using Evidential Regression (EVREG) and a discount function based on the contribution of each dimension. The method “learns” the optimal weights for each feature using a gradient descent technique. The method also uses the nearest k-neighbor algorithm to accelerate calculations. It is possible to select the most relevant features for predicting a patient’s health care costs using this approach and the transparency of the Evidential Regression model. We can obtain a reason for a prediction with a k-NN approach. We used the Japanese health records at Tsuyama Chuo Hospital to test our method, which included medical examinations, test results, and billing information from 2013 to 2018. We compared our model to methods based on an Artificial Neural Network, Gradient Boosting, Regression Tree and Weighted k-Nearest Neighbors. Our results showed that our transparent model performed like the Artificial Neural Network and Gradient Boosting with an $R^{2}$ of 0.44.

## 1. Introduction

Health care expenditure is one of the most critical issues in today’s society. World Health Organization (WHO) statistics show that global health care expenditure was approximately US$ 7.5 trillion, equivalent to 10% of the global GDP in 2016 [1]. One of the reasons for these high expenses in care is the low accountability in health care in some countries, for example, inefficiencies in the US health care system result in unnecessary waste, provoking a large discrepancy between spending and returns in care [2]. If we could predict health care costs for each patient with high certainty, problems such as accountability could be solved, enabling control over all parties involved in patients’ care. It could also be used for other applications such as risk assessment in the health insurance business, allowing competitive insurance premiums, or as input information for developing new government policies to improve public health.

With the current frequently used electronic health records (EHRs), an interest has emerged in solving accountability problems using data mining techniques [3]. There have been various approaches to predict health care costs for large groups of people [4,5]. On the contrary, prediction for an individual patient has rarely been tackled. Initially, rule-based methods [6] were used for trying to solve these problems requiring domain knowledge as if-then rules. The downside of this method is the requirement of a domain expert to create the rules, thus making the solution expensive and limited to the dataset being used. In the current state-of-the-art, statistical and supervised learning methods are preferred with the latter getting the best performance. The reason for best performance is the skewed and heavy right-hand tail with a spike at zero present in the distribution of health care costs [7].

Supervised learning methods can be evaluated by performance and interpretability; usually, the most sophisticated methods are the ones that have best performance, sacrificing interpretation (e.g., Random Forest, Artificial Neural Networks, and Gradient Boosting). A drawback of these high performing machine learning algorithms in health care is their black-box nature, especially in critical use cases. Even though health care cost prediction is not a critical use case, using patients’ personal and clinical information for this problem could suffer biased results without an interpretable method. Interpretable methods would allow patients, physicians, and insurers to understand the rationale behind a prediction, giving them the option to accept or reject the knowledge the method is providing.

The aim of this work is to create an intrinsic interpretable regression method for health care costs prediction, with a performance comparable to state-of-the-art methods, inspired by the work of Peñafiel et al. [8], where an interpretable classifier based on the Dempster–Shafer Theory (DST) [9] was presented. The Dempster–Shafer Theory (DST) [9] is a generalization of the Bayesian theory. It is more expressive than classical Bayesian models since it allows us to assign “masses” to multiple outcomes measuring the degree of uncertainty of the process. We could have extended the work of the interpretable classifier but as it is a pure classification algorithm, the output is assumed to be discrete, and some procedures do not apply to continuous outputs, e.g., the time complexity grows exponentially with the number of classes. Petit-Renaud and Denux [10] introduced the use of Dempster–Shafer theory for regression problems; they presented a regression model that uses DST called Evidential Regression (EVREG) to find the expected value of a variable using a set of examples as evidence. We will extend EVREG using a weighted distance and gradient descent, which enables the model to do feature selection (FS) tasks and use it in the health care costs prediction problem. The  weight of each feature will represent the importance of this feature to predict an outcome in a dataset; thus, a set of masses with these weights will be computed; the masses will represent the importance of samples in the training set to predict an outcome.

Previous works in health care costs prediction [11,12,13] have reached to the conclusion that clinical features yield the same performance as using only cost predictors without the proper FS experiment. One aim of our work is to prove these claims with a proper FS method. Our research question is whether it is possible to develop an interpretable method with FS capabilities that has a similar performance to black-box methods for the health care cost prediction problem. We used Japanese health records from Tsuyama Chuo Hospital to test our answer. These records include medical checkups, exam results, and billing information from 2013 to 2018. We used them to compare our method performance with the performance obtained by less interpretable methods such as Artificial Neural Networks (ANNs) and Gradient boosting (GB).

First, we will describe the state-of-the-art for health care cost predictions, introduce FS and DST. Then we will describe our proposed model, which we named Weighted Evidential Regression (WEVREG) with its implementations and improvements. Finally, we will present the model performance in feature selection tasks against prediction methods with FS capabilities using synthetic data, besides its performance in health care costs prediction.

Our results show that WEVREG is able to perform FS tasks obtaining better performance for features with complex dependencies. In health care costs prediction, our method outperforms ANN, and gets similar results to GB but cannot reach the same performance. The features selected by our method show that cost variables are the most important ones to predict future costs, confirming the results obtained in previous works.

This paper extends the work presented previously in [14] by improving the prediction algorithm and showing how this model can perform feature selection. The paper also explains which are the features in the input data that are most important for making the prediction of the health costs.

## 2. Related Work

### 2.1. Health Care Cost Prediction

Health care costs commonly have a spike at zero and a skewed distribution with a heavy right-hand tail; statistical methods (e.g., linear regression) suffer from this characteristic in small to medium sample sizes [15]. Advanced methods have been proposed to address this problem, for example, Generalized Linear Models (GLMs) where a mean function (between the linear predictor and the mean) and a variance function (between the mean and variance on the original scale) are specified and the parameters are estimated given these structural assumptions [16]. Another example is the two-part and hurdle model, where a Logit or Probit model is used in the first instance to estimate the probability of the cost being zero, and then if it is not, a statistical model is applied, such as log-linear [17] or GLM. The most complex statistical method used to solve this problem is the Markov chain model; an approach based on a finite Markov chain suggested estimating resource use over different phases of health care [18]. Mihaylova et al. [19] present a detailed comparison of statistical methods in health care cost prediction.

Supervised learning methods have been extensively used to predict health care costs; the data used for these methods vary. While a few works use only demographic and clinical information (e.g., diagnosis groups, number of admissions and number of laboratory tests) [20], the majority have incorporated cost inputs (e.g., previous total costs, previous medication costs) as well [11,12,13,21], obtaining better performance. GB [22] excels as the method with the best performance for this problem [13], which is an ensemble-learning algorithm, where the final model is an ensemble of weak regression tree models, which are built in a forward stage-wise fashion. The most essential attribute of the algorithm is that it combines the models by allowing optimization of an arbitrary loss function, in other words, each regression tree is fitted on the negative gradient of the given loss function, which is set to the least absolute deviation [23]. ANNs come close to the performance of GB. An ANN is an extensive collection of processing units (i.e., neurons), where each unit is connected with many others; ANNs typically consist of multiple layers, and some goal is to solve problems in the same way that the human brain would do it [24]. Another type of model with good results is the M5 Tree [12]; this algorithm is also a Regression Tree (RT), where a Linear Regression Model is used for building the model and calculating the sum of errors as opposed to the mean [25].

Most health care expenses of a population are generated by a small group, as Bertsimas et al. [11] showed in their dataset: 80% of the overall cost of the population originates from only 20% of the most expensive members. Therefore, a classification phase is suggested to classify patients in a risk bucket when trying to improve the performance of the methods listed above. Morid et al. [7] reported that for low-risk buckets, GB obtains the best results, but for higher ones, ANN is recommended. It has also been found that costs rise sharply with nearness to death [26,27,28]. This fact needs to be taken into account when building the embedding for the input vectors of a health care costs dataset.

### 2.2. Feature Selection

Nowadays, there are several datasets with high dimensionality. Reducing the number of features while maintaining the models performance has become indispensable. This strategy decreases the dimensionality of the Euclidean space, therefore preventing the curse of the dimensionality phenomenon that causes a drop on models accuracy [29]. Feature selection (FS) is the ability to select the features with the highest correlation to the target variable [30]; in particular they select a subset of features based on a certain evaluation criteria. Some features that do not meet the criteria are eliminated; the goal is to eliminate redundant or irrelevant features that are a priori unknown. This strategy decreases the number of dimensions, often resulting in an increase of models accuracy and time performance. FS has been of great importance in areas such as DNA microarray analysis, text categorization and information retrieval [31,32]. FS methods can be classified in three types: filter, wrapper and embedded methods [33]. Filter methods are typically the simplest and cheapest to compute; they apply statistical techniques to test the correlation of each feature with the target variable: normally a threshold is set to choose the most relevant features. The most well-known filter methods include Pearson’s correlation coefficients [34] which assigns a value to two variables, between −1 and 1 representing the linear dependency between them. F-score [35] is another example, where the weighted harmonic mean of the test precision and recall is computed varying from 0 to 1. The disadvantage of these methods is that they cannot detect complex relations as they do not reveal mutual information between features. Wrapper methods are expensive because they test multiple subsets of features in a prediction model and select the subset maximizing the performance of the prediction. The most well-known method for regression problems is the Linear Support Vector Regressor (L1-SVR) [36] which given a toleration error, finds the hyper-plane that maximizes the margin between vectors. Feature importance is then obtained from the SVR coefficients. Embedded methods, unlike the previous methods, include the feature selection process as their learning phase. Common embedded methods include various types of decision tree algorithms. Some of the most popular ones are the Random Forest (RF) [37]—a method which uses multiple decision trees to reach an outcome—and Gradient Boosting (GB) [38], where weak decision trees are built, and features are selected sparsely following an important change in the impurity function.

Since FS is an important pre-processing step in most machine learning applications, it has been widely studied with new methods constantly appearing. There has been recent interest in FS algorithms based on k-neighbors. ReleifF [39] is a filtering method that randomly samples an instance of the data and locates its *k* nearest neighbor, the *k* instances are then used to update the score of each feature. Other recent approaches, Regression Gradient Guided Feature Selection (RGS) [40] and Weighted Nearest Neighbors(WkNN) [41] are methods that use a Weighted k-NN model with a gradient descent as an optimization approach to find the optimal weight vector used in the k-NN distance function. These two algorithms differ in the gradient descent algorithm and discount function used to find the optimal importance of each feature.

### 2.3. Dempster–Shafer Theory

Let *X* be the set of all states of a system called frame of discernment. A mass assignment function *m* is a function satisfying:

(1) $$m:2^{X}\to \left[0,1\right],\;\;\;m\left(\phi \right)=0,\;\;\;\sum_{A\subseteq X}m\left(A\right)=1$$

The term m(A) can be interpreted as the probability of getting precisely the outcome *A*, and not a subset of *A*.

Multiple evidence sources expressed by their mass assignment functions of the same frame of discernment can be combined using the Dempster Rule (DR) [42]. Given two mass assignment functions $m_{1}$ and $m_{2}$, a new mass assignment function $m_{c}$ can be constructed by the combination of the other two using the following formula:

(2) $$\begin{array}{cc} m_{c}\left(A\right) & =m_{1}\left(A\right)⊕m_{2}\left(A\right)\\ \; & =\frac{1}{1-K}\sum_{B\cap C=A\neq \phi }m_{1}\left(B\right)m_{2}\left(C\right) \end{array}$$

where *K* is a constant representing the degree of conflict between $m_{1}$ and $m_{2}$ and is given by the following expression:

(3) $$K=\sum_{B\cap C=\phi }m_{1}\left(B\right)m_{2}\left(C\right).$$

Petit-Renaud and Denux [10] introduced a regression analysis algorithm based on a fuzzy extension of belief functions, called evidence regression (EVREG). Given an input vector **x**, they predict a target variable **y** in the form of a collection of evidence associated with a mass of belief. This evidence can be fuzzy sets, numbers, or intervals, which are obtained from a training set based on a discount function that takes their distance to the input vector **x** and is pooled using the Dempster combination rule (Equation (2)). They showed that their methods work better than similar standard regression techniques such as the Nearest Neighbors using data of a simulated impact of a motorcycle with an obstacle.

The EVREG model has been used for predicting the time at which a system or a component will no longer perform its intended function (machinery prognostic) for industrial application. Niu and Yang [43] used the EVREG model to construct time series, whose prediction results are validated using condition monitoring data of a methane compressor to predict the degradation trend. They compared the results of the EVREG model with six statistical indexes, resulting in a better performance of the EVREG model. Baraldi et al. [44] used the model to estimate the remaining useful life of the equipment. Their results have shown the effectiveness of the EVREG method for uncertainty treatment and its superiority over the Kernel Density Estimation and the Mean-Variance Estimation methods in terms of reliability and precision.

## 3. Data and Problem Description

The problem we address in this work is predicting future health care cost of individuals, using their past medical and cost information. This is a supervised learning problem, which can be formally specified as a regression problem where the input vector $x=(x_{0},x_{1},\cdots ,x_{n})$ is an individual’s past medical and cost information and the target variable *y* is that person’s health care expenses in a future period (e.g., a year).

The records used for this work were provided by Tsuyama Chuo Hospital, a Japanese hospital located in Okayama prefecture. These records were gathered between 2013 and 2018.

Japan has universal coverage for social health insurance; the system is composed of three sub-systems, National Health Insurance (self-employment), Society Health insurance (for employees) and a Special Scheme for the elderly (75 or older). Every citizen must join one of these three sub-systems according to their occupational status and age. The premium charge for each person is set by each insurer depending on the person’s income. Each medical organization is paid by a fee-for-service principle; at the end of the month every medical facility in Japan has to send a set of claims to be reimbursed by an insurer (as a claim sheet); the insurers have the right to decline a claim if it is incorrect or seems unnecessary.

Medical facilities use a special software for the production of a claim sheet. This software registers all procedures, drugs and devices for each patient. Each procedure has a standard code set by the Ministry of Health, Labor and Welfare (MHLW) that can be translated directly to the International Statistical Classification of Diseases and Related Health Problems codification (ICD-10) [45], a medical classification list created by the World Health Organization (WHO).

Every claim sheet send by health facilities all over Japan are gathered by the MHLW in a National Database [46]. The database contains a detailed information on patients such as provided service, age, sex, date of consultation, date of admission, date of discharge, procedures and drugs provided with volumes and tariffs. 1700 million records are registered annually. Unfortunately, we do not have access to the National Database, but we have access to the electronic claims (claim sheets) sent by Tsuyama Chuo hospital to the National Database between 2013 and 2018.

The claims are stored in a set of files; we had to transform these files to study the data. The format of the claims file is confusing without previous knowledge of the structure. The detailed documentation of this format can be found at the Medical Remuneration Service website (http://www.iryohoken.go.jp/shinryohoshu/file/spec/22bt1_1_kiroku.pdf). A short summary of the file format is shown in Table 1.

Since a patient’s data are dispersed within these files, we could not use these raw data to predict the health care expenditure of patients. We used the data in these files to create a patient’s representation that we could use for the prediction of an individual’s health care costs. Each patient in the insurance claims could be identified by a unique identifier, which enabled us to follow all patients throughout the years.

We had access to patients’ health checkups as well as to the data sent by Tsuyama Chuo hospital to the National Database. Every Japanese worker needs to take a yearly health checkup to start or continue working at a company, so there were many patients with health checkup data.

Our dataset had patients’ monthly history between 2013 and 2018. However, there were many missing values because most patients had few claims each year. Therefore, we grouped claims yearly so that we could have fewer missing values. We created three different scenarios for this purpose as follows. **Scenario 1**: We used a single year of history to predict the next cost value. **Scenario 2**: We used two years of data to predict the third one, and **Scenario 3**: We used five years of history to predict the costs of the sixth year. We filtered out patients in each scenario if they did not have the required history available, and thus, the sets shrank in each scenario. In the case of missing health check values, we opted to use the median of the exam to replace null values. Table 2 shows the basic statistics of the sets in each scenario.

## 4. Proposed Model

A regression task predicts the value of a target variable *y* using as input an arbitrary vector *x*. For a regression method to succeed at this task, the predicted target variable value $\hat{y}$ needs to be as close as possible to the real target variable *y* value. In particular, in a Supervised Learning problem, variable $\hat{y}$ is deduced from a training set or the set of examples that are taken from past observations. In summary a regression task solves the problem of finding a function f(x) which best explains the target variable *y*.

Petit-Renaud and Denœux presented a regression method based on the Dempster–Shafer Theory (DST) called Evidential Regression (EVREG) [10]. The EVREG model uses a set of observations that have occurred in the past as evidence in order to predict the target value of a new observation. Each observation in this evidence set is given a mass which represents the similarity of the new observation with each observation in the set. We calculate this similarity using a distance function (e.g., Euclidean distance) in the feature space of the observations. The DST ensures that the masses of this evidence set add up to 1, so they can be easily transformed to a probability distribution. Then an expected value is computed as the mass of each past observation $m_{i}$, times its observed target value ($y_{i}$), as shown in Equation (4).

(4) $$E\left[y\right]=\sum_{i=1}^{N}m_{i}*y_{i}$$

DST is characterized for reasoning with uncertainty. EVREG uses the width of the observed target variable interval (difference between maximum and minimum target variables) to represent uncertainty as another piece of evidence. The importance given by the model to this interval represents the uncertainty the model has when predicting an outcome. For example, when the new observation is at a great distance from the evidence set, the model assigns a high value to the evidence of the variable *y* interval; thus we get a high uncertainty in the model outcome, resulting in upper and lower bounds for the predicted target variable proportional to the interval of the target variable observed in the evidence set.

EVREG has been used for predicting the time at which a system or a component will no longer perform its intended function (machinery prognosis) for industrial applications [43,44]. Niu and Yang [43] used the EVREG in a time series task, to predict the degradation trend of a methane compressor. They compared the results of the EVREG model with six statistical indexes; the results showed the EVREG model had the best performance. Baraldi et al. [44] used the model to estimate the remaining useful life of industrial equipment. Their results have shown the effectiveness of the EVREG method for uncertainty treatment and its superiority over the Kernel Density Estimation and the Mean-Variance Estimation methods in terms of reliability and precision.

We will first describe EVREG (Section 4.1) in this section. Then, its time complexity and time improvement will be explained in Section 4.2. We will extend EVREG using gradient descent and a weighted distance function in Section 4.3 and Section 4.4; the goal will be to improve the model regression performance and enable it to perform feature selection tasks, which will allow it to rank features in a space. Using this new distance function and optimization approach, the model will update the weight of each dimension. The extended model will be able to rule out or assign low weights to irrelevant features, and assign higher values to the ones that strongly influence the output. This is an important characteristic since feature selection has been shown to improve model accuracy and computing times [40,41,47]. Our aim is that this enhanced EVREG method will perform as well as state-of-the-art regressors for tabular and time series regression problems. In addition, the model will possess a feature selection ability, ranking the features representing the correlation of each feature with the target variable, enabling its users to gain more knowledge of the used data.

### 4.1. Evidential Regression

EVREG [10] is a Supervised Learning algorithm. This kind of algorithm uses a sample set as examples to make a prediction. The samples in a Supervised Learning problem are called the training set. Formally, we define the training set as:

(5) $$L:{\left\{e_{i}=\left(x_{i},y_{i}\right)\right\}}_{i=1}^{N}$$

where $e_{i}$ is an element of the training set, $x_{i}$ is the input vector of $e_{i}$ and $y_{i}$ its output or target value.

Let *x* be an arbitrary vector and *y* its corresponding unknown target variable. We need to derive the expected value $\hat{y}$ of variable *y* from the training set L. Each element $e_{i}$ of the training set is a piece of evidence concerning the value of *y*. The relevance of each element in L can be assumed to depend on the similarity between the arbitrary vector *x* and the input vectors $x_{i}$ of $e_{i}$. It can be assumed that a suitable discount function that depends on a distance measure ∥·∥ can measure this similarity. If *x* is close to a vector $x_{i}$ in L, we assume that *y* is also close to $y_{i}$, which makes the element $e_{i}$ an important piece of evidence. If *x* and $x_{i}$ are at a great distance, it is safe to assume that $y_{i}$ has a small effect on *y*, and provides only marginal information concerning *y*. We will use the Minkowski distance defined as:

(6) $$d\left(x_{i},x_{j}\right)=(\;\sum_{k=1}^{l}|x_{ik}-x_{jk}{{|}^{p}\;)}^{\frac{1}{p}}$$

where $x_{ik}$ and $x_{jk}$ are the values of vectors $x_{i}$ and $x_{j}$ at dimension *k*. When *p* is 1 we get the L1 or Manhattan distance and with *p* equal to 2 we get L2 or also known as the Euclidean distance. In the case of high dimensional spaces, the vectors become uniformly distant from each other, the ratio between the nearest and farthest vector approaches 1. This phenomenon is more present in the Euclidean than in the Manhattan distance metric [48,49], which makes the Manhattan distance to yield better results in distance-based algorithms in the presence of a high dimensional space. EVREG could use any distance measure (e.g., cosine distance) to represent the similarity between two vectors. We will use the Minkowski distance for its flexibility while testing the performance for values p∈[1,2]. The value of *p* will be chosen as the one that yields the best prediction performance in each problem. We will define the discount function ϕ between vectors $x_{i}$ and $x_{j}$ using this distance measure as:

(7) $$\phi \left(d\left(x_{i},x_{j}\right)\right)=\exp\left(-{\frac{d(x_{i},x_{j})}{\gamma }}^{2}\right)$$

where ϕ is a decreasing function from IR to [0,1] that needs to fulfill ϕ(0)∈[0,1] and,

(8) $$\lim_{d\to \infty }\phi \left(d\right)=0$$

**Figure 1:**
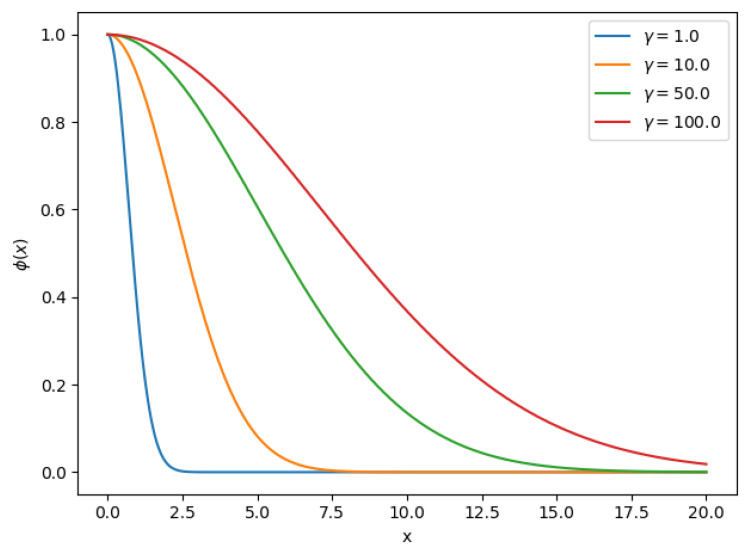
Radial Basis Function for different γ values.

Equation (7) is the well-known Radial Basis Function (RBF) that decreases monotonically with distance, commonly used as a kernel in the Support Vector Machine (SVM) classifier. Parameter γ is the radius of the function, intuitively γ defines how far the influence of a vector reaches. In Figure 1 we can observe Equation (7) for different γ values. RBF-based methods are an active area of research [50,51]; various approaches exist to find the optimal parameters such as γ. This parameter can be learned using an optimization approach, but often the value is set manually by trial and error. We will try to find the optimal γ using a Grid Search approach in this work

The discount function ϕ will represent the similarity between two vectors (higher value means higher similarity); we can use this similarity function to compute the mass (influence) of each element in L given an arbitrary vector *x* using the Dempster rule of combination getting:

(9) $$m_{i}\left(x\right)=\frac{1}{K}\phi \left(d\left(x,x_{i}\right)\right)\prod_{k!=i}\left(1-\phi \left(d\left(x,x_{k}\right)\right)\right)$$

where *K* is a normalization term defined by the DST as:

(10) $$K=\prod_{j=1}^{N}\left(1-\phi \left(d\left(x,x_{i}\right)\right)\right)+\sum_{i=1}^{N}\left[\phi \left(d\left(x,x_{i}\right)\right)\prod_{k!=i}\left(1-\phi \left(d\left(x,x_{k}\right)\right)\right)\right]$$

In Equation (9), ϕ determines the influence of $x_{i}$ in *x* and the product determines the effect of the evidence set between these two vectors. We can obtain the influence of every vector in set L using the previous formulas, but we need to consider one extra piece of evidence. We have observed the values of variable *y* in every example of set L. EVREG takes this information into account. We will assume that variable *y* is bounded to the interval $\left[y_{inf},y_{sup}\right]$, and for this interval we will define an additional mass called domain mass $m^{*}$ calculated as:

(11) $$m^{*}\left(x\right)=\frac{1}{K}\prod_{i=1}^{N}\left(1-\phi \left(d\left(x,x_{i}\right)\right)\right)$$

Since we assumed that *y* is bounded to the $\left[y_{inf},y_{sup}\right]$ interval, there exists a probability density function P(x) associated to $m_{i}\left(x\right)$ and $m^{*}\left(x\right)$. Smets et al. [52] showed that the Pignistic transformation could be used to transform the masses in EVREG to a probability function. In the particular case where output *y* is a real number, the Pignistic probability is defined as:

(12) $$P\left(x\right)=\sum_{i=1}^{N}m_{i}\left(x\right)\cdot \delta_{i}\left(x\right)+\frac{m^{*}}{y_{sup}-y_{inf}}$$

Equation (12) is a mixture of Dirac distributions and a continuous uniform distribution. With this probability function, we can get the expected value of target variable *y* as the Pignistic expectation [10]:

(13) $$\hat{y}=\sum_{i=1}^{N}m_{i}\left(x\right)\cdot y_{i}+\frac{m^{*}\left(x\right)\cdot \left(\underset{y\in L}{\sup}y+\underset{y\in L}{\inf}y\right)}{2}$$

where $\hat{y}$ is the expected or predicted value of target variable *y*. With the Pignistic expectation we have an upper and lower bound for variable $\hat{y}$ as:

(14) $${\hat{y}}^{*}=\sum_{i=1}^{N}m_{i}\left(x\right)\cdot y_{j}+m^{*}\left(x\right)\cdot \underset{y\in L}{\sup}y$$

(15) $${\hat{y}}_{*}=\sum_{i=1}^{N}m_{i}\left(x\right)\cdot y_{j}+m^{*}\left(x\right)\cdot \underset{y\in L}{\inf}y$$

We can observe that the interval $\left[{\hat{y}}_{*},{\hat{y}}^{*}\right]$ contains variable $\hat{y}$. The width of this interval can be interpreted as the uncertainty of the response, which is directly associated with the mass of the domain of target variable *y*.

The computation time of a prediction grows quickly with the size of the training set. For a single prediction in training set L of size *n*, we need to compute the mass of each element $e_{i}$ which has a vector $x_{i}$ of dimension *q*. We start by pre-computing the discount function (ϕ) of the input vector *x* with every element $e_{i}$ in the set L. This computation takes O(q) time for each element in the set, taking a total time of O(nq) to compute each mass of L additionally to the discount function. In Equation (9) we need to compute a product sequence and the normalization term *K*. As we have pre-computed the discount functions for the training set, the product sequence takes only O(log(n)). As for the normalization term *K* (Equation (10)) which takes most of the calculation time for computing Equation (9), the product sequence on the left side can also be computed in O(log(n)). The sum of the right side takes time O(nlog(n)) because of the product sequence contained in it. The domain mass also requires the normalization term *K* and the product sequence takes time O(nlog(n)). Thus the time complexity for a single mass in the training set is O(nlog(n)).

### 4.2. Improving Computing Time

All the masses of the training set need to be calculated to compute a single prediction. The mass calculation needs to be computed *n* times; since we compute a single mass in time O(nlog(n)), the *n* masses will take time $O(n^{2}log\left(n\right))$. Computing a prediction with the formula shown in Equation (13) uses the masses of the set L, and the mass of the domain of target variables in the training set. This calculation requires to compute the maximum and minimum values, which needs time O(n), so we can get the prediction of input vector *x* in time $O(n^{2}log\left(n\right))$.

The quadratic growth of each prediction makes it impossible for the EVREG model to work with large sets; therefore it is difficult to apply it for real-life problems. However, it has been suggested the use of a k-Nearest Neighbors (k-NN) approach [10] to improve computation time. Their results showed that a k-NN approach did not introduce a significant penalty to the algorithm performance. It is possible to create indexes for the k-NN search in O(n(q+k)) with this approach in the following way. First, we compute the distance between *x* and $x_{i}$ for each vector in the training set, then we iterate through the distances *k* times selecting the smallest distance. Now, only the masses of the *k* nearest neighbors have to be calculated when computing a new prediction of a vector *x*. The masses of samples that are not in the set of the nearest neighbors of *x* will be assumed to be null. In particular, we use a flat index implementation by Johnson et al. [53], for the exact nearest neighbor’s search given its better execution times and memory usage compared to other existing solutions.

Given now that only the masses of the *k* Nearest Neighbors are relevant for a prediction, the mass of each sample in the training set for an input vector *x* is calculated as:

(16) $$m_{i}\left(x\right)=\left\{\begin{array}{cc} \frac{1}{K}\phi \left(d\left(x,x_{i}\right)\right)\prod_{\begin{array}{c} k!=i\\ x_{k}\in N\left(x\right) \end{array}}\left(1-\phi \left(d\left(x,x_{h}\right)\right)\right) & \mathrm{if}x_{i}\in N\left(x\right),\\ 0 & \mathrm{otherwise} \end{array}\right.$$

where N(x) is the set of *k* Nearest Neighbors of vector *x*. Now the normalization term *K* can be computed as:

(17) $$K=\prod_{x_{i}\in N\left(x\right)}\left(1-\phi \left(d\left(x,x_{i}\right)\right)\right)+\sum_{x_{i}\in N\left(x\right)}\left[\phi \left(d\left(x,x_{i}\right)\right)\prod_{\begin{array}{c} h!=i\\ x_{h}\in N\left(x\right) \end{array}}\left(1-\phi \left(d\left(x,x_{h}\right)\right)\right)\right]$$

Just the *k* neighbors are considered as evidence for a prediction of the domain mass, so the domain evidence is only attributed to its neighbors. We compute the calculation of the domain mass as:

(18) $$m^{*}\left(x\right)=\frac{1}{K}\prod_{x_{i}\in N\left(x\right)}\left(1-\phi \left(d\left(x,x_{i}\right)\right)\right)$$

Since now we compute only the masses of the *k* Nearest Neighbors, we have to change the size of the training set *n* by the number of neighbors *k* instead in the expression for computing the time complexity. In Equation (16) once we got the *k* Nearest Neighbors, the normalization term *K* will be computed in O(qklog(k)). The discount function will still take O(q) and the product sequence will have O(qklog(k) complexity. Only the masses of the *k* neighbors will be needed to compute a prediction, so only *k* masses of the training set will be computed thus obtaining a complexity for a single prediction of $O(qk^{2}log\left(k\right))$. Assuming that *k* is always significantly less than *n*, the conclusion is that the complexity for a prediction ends up being reduced to O(nqk) because of the complexity of the k-NN search.

**Figure 2:**
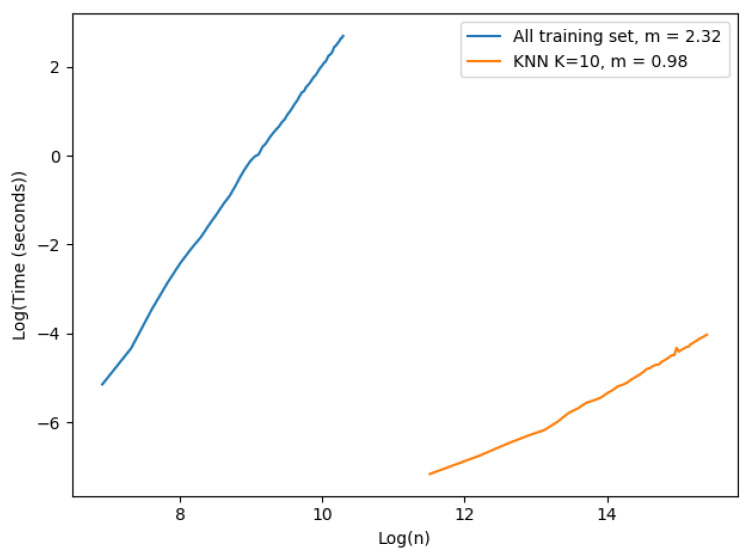
Time complexity single prediction.

We performed the following experiment to demonstrate the importance of the k-NN approach for speeding up the prediction computing time. We had two different settings, one for each approach: (i) the first approach used all the training set as evidence; we started with 1000 examples and ended at 30,000 with steps of size 500 in this case. (ii) the k-NN approach; the examples started at 100,000 and finished at 5,000,000 examples with steps of size 100,000. We fixed the number of neighbors to 10 and the number of dimensions used was only 1. The results are shown in Figure 2. Each value shown in the figure corresponds to the mean execution time of 100 experiments.

The plot in Figure 2 is in a logarithmic scale. The slope of each curve was computed to observe the complexity of each approach. The *x* axis corresponds to the logarithmic number of samples in the training set, and the *y* axis is the logarithmic execution time of a single prediction in seconds. The number of vectors varied in each approach due to hardware constraints: case (i) we had to limit the number of vectors that could be tested because of excessive execution time and memory consumption. Case (ii) the machine where the experiment ran could not register execution times properly for a low number of vectors in the training set. As expected, the time complexity of the implemented EVREG with no optimization grew dramatically faster than the k-NN approach as seen by their slopes. The complexity of using all the training set approach seemed to have a larger time complexity than theoretically expected, as the slope in a complexity of $O(n^{2}log\left(n\right))$ was 2.12. In the k-NN approach we could easily observe a linearity with the size of the training set. Consequently, it became obvious that the k-NN approach significantly dropped execution times and made EVREG a viable option for real world problems.

### 4.3. Weighted Evidential Regression

This section proposes an improvement to the discount function used in EVREG based on ideas which has been previously introduced to enhance the well-known k-Nearest Neighbors Regressor (k-NN Regressor) [54], which is another regressor, similar to EVREG. The improved model will be called Weighted Evidential Regression (WEVREG) Model. The aim of this improvement is to boost prediction performance and allow the model to perform feature selection tasks. The k-NN Regressor is a simple and intuitive non-parametric regression method to estimate the output value of an unknown function for a given input. It “guesses” the output value using samples of known values as a training set, and computing the mean output value of the nearest neighbors of the input vector. All neighbors in this method are equally relevant for predicting the target variable in its original version. There is a variation of this algorithm where the importance of each neighbor depends on a distance measure between them, thus using a weighted average of the k-NN vectors in the training set. This weighted k-NN Regressor is a kind of locally weighted regression [55]. The weight of each neighbor is proportional to its proximity to the input vector. The prediction is computed with this approach by the following expression:

(19) $$\hat{f}\left(x\right)=\frac{1}{Z}\sum_{x^{\prime }\in N\left(x\right)}f\left(x^{\prime }\right)\;e^{\frac{-d(x,x^{\prime })}{\beta }}$$

where N(x) is the set of *k* Nearest Neighbors of vector *x*, f(x’) is the value of the target variable of neighbor x’, d(x,x’) is a distance function between vector *x* and its neighbor x’ (commonly the Euclidean distance), β is a parameter of the estimator and *Z* is a normalization function with value $Z=\sum_{x’\in N(x)}e^{\frac{-d(x,x’)}{\beta }}$.

Similar to the EVREG, the weighted k-NN regression uses the Euclidean distance and a Gaussian Radial Basis discount function to assign a weight to each one of its neighbors. Further improvements have been made to the weighted k-NN Regression based on the assumption that the target variable is most accurately predicted using only the most relevant features of the neighbors which adds a Feature Selection process to the algorithm. Navot et al. [40] introduced a weighted distance function for the weighted k-NN Regressor, which improved the model performance and enabled the model to perform a feature selection task. Given a weights vector *w* over the features of size *q* the distance function induced by *w* is defined as:

(20) $$d\left(x_{i},x_{j}\right)=(\;\sum_{k=1}^{l}|\left(x_{ik}-x_{jk}\right)\cdot w{{|}^{p}\;)}^{\frac{1}{p}}$$

where input vector $x_{i}$ and $x_{j}$ have the same size *q* as the weights vector *w*. Each value of vector *w* represents the importance for each dimension in computing the distance between two vectors, i.e., the amount which a feature contributes to the distance between these two vectors. The model assumes that every dimension contributes the same to the distance between two vectors in EVREG Equation (6), and consequently, to our discount function (Equation (7)). However, this is not always the case; for example we could have a feature in our input that has no impact in the target variable we are trying to predict thus it contributes nothing to the similarity between those vectors. For instance, a patient age could have a great impact on that individual health care costs so to predict the costs we want to be closer to patients in the same age group, but maybe the eye color does not influence this cost, so we would not want to include two patients with the same eyes color just for this fact.

We will change the distance function in Equation (6) with the one that uses weights as presented in Equation (20) in order to improve the prediction performance of the EVREG model and to gain further understanding about the data.

We will also use the distance measure described in Equation (20), to compute the k-NN of each input vector *x*. The k-NN search implementation we use does not have a feature to use a custom distance measure to compute a k-NN search, so we will have to create the indexes with a transform training set space (applying the weights vector), and then transform the input vector *x* that will be predicted. We will get the same distance measure as described in Equation (20) with this operation.

**Figure 3:**
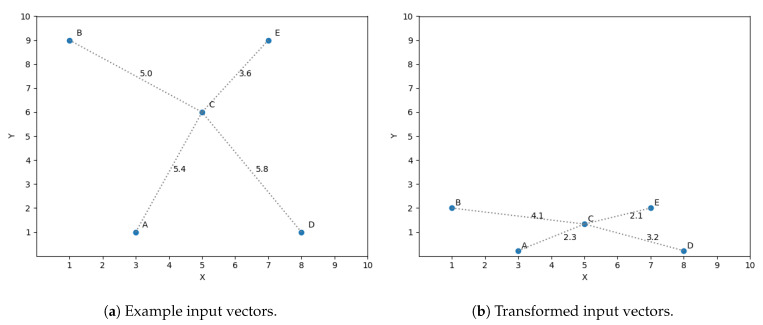
Feature transformation.

A simple example is presented to ease the understanding of the chain of thoughts behind this idea. Let us assume we have a sample set of size 5 in a two dimensional space and we want to predict its third dimension. The input vectors (two dimensions) are shown in Figure 3a. The optimization discussed in Section 4.2 is used in this example, taking into account only the three closest neighbors to predict the target variables. If we want to predict the output value for vector *C* using the three closest neighbors, then we should consider *A*, *B* and *E* as its closest neighbors, shown in Figure 3a by the distance between *C* and all the vectors. Nevertheless, what if we find that the dimension *y* is not as significant as *x* for predicting the target dimension? This observation means a variation in *y* does not affect in the same magnitude the prediction as a difference of the same size in *x*. The space to reflect the variation in magnitude is scaled, getting Figure 3b.

If we now compute the three closest neighbors for vector *C* then we get vector *A*, *E* and *D*, replacing vector *B*, thus reflecting the importance of feature *x* in the similarity function.

If we want the weight of a feature to represent the importance in an input vector, then the range of each one of the feature domains has to be of the same size. Otherwise, we could get a bigger weight of a dimension only to compensate the size of the domain. Therefore, it is recommended to normalize the input features (all values must be between 0 and 1) so that the weights are comparable among features.

We can find the optimal weight vector using an optimization approach such as gradient descent. We will describe the process of finding the optimal weights for a known training set in the next subsection.

### 4.4. Weight Learning

This subsection will present the process of finding the optimal weights vector in a known training set. We will use a gradient descent algorithm to find a vector *w* that minimizes the error of the prediction model.

The accuracy of a regressor is commonly measured by computing the difference between the predicted value $\hat{Y}$ and the actual value of the target variable *Y*. The estimation error is expressed by a loss function L(*Y*, $\hat{Y}$), L:IR×IR→IR. Likewise in a Supervised Learning problem, our goal is to find a vector *w* that induces the smallest error in *L* using the training set L and a small generalization error in a validation set at the same time. We will use a gradient-based algorithm such as gradient descent with an Adam optimizer to get the optimal value for *w*, because of its clear mathematical justification for reaching optimum values [56].

The gradient descent algorithm is an iterative algorithm that optimizes variable values in order to find a function minimal value. We use the Mean Squared Error (MSE) as our error function [57], and we define the loss function *L* induced by *w* as:

(21) $$L_{w}\left(Y,\hat{Y}\right)=\frac{1}{n}\sum_{i=1}^{n}{\left(y_{i}-\hat{y_{i}}\right)}^{2}$$

where *n* is the number of samples in the training set, *Y* is a vector of size *n* with the actual values for the target variable of each sample in the training set, and $\hat{Y}$ is the predicted value for each one of the samples in the training set. *y* and $\hat{y_{i}}$ are elements of *Y* and $\hat{Y}$ respectively.

After defining the function error *L* which is induced by the weights vector *w*, our goal is to find a vector *w* that yields the smallest error possible for the training set given. The estimator $\hat{w}$ for vector *w* is obtained by minimizing this criterion:

(22) $$\hat{w}=argmin\;L_{w}\left(Y,\hat{Y}\right)$$

Since our loss function $L_{w}$ is continuous and differentiable, we can use gradient descent algorithm to find $\hat{w}$. The gradient of $L_{w}$ needs to be computed in every iteration of our algorithm in order to update the weights *w*, then *w* is updated by taking a step proportional to the negative of the gradient. We need the partial derivation of $L_{w}$ for the gradient computation. This derivation is calculated as:

(23) $$\frac{\partial L_{w}}{\partial w}=\frac{2}{n}\sum_{i=1}^{n}\frac{\partial (y_{i}-\hat{y_{i}})}{\partial w}=\frac{2}{n}\sum_{i=1}^{n}\frac{\partial (y_{i}-\hat{y_{i}})}{\partial \hat{y_{i}}}\cdot \frac{\partial \hat{y_{i}}}{\partial w}$$

We need to calculate the partial derivative of the predicted target variable $\hat{Y}$ with respect to *w* in order to solve Equation (23). Therefore, the calculation of the derivative of Equation (13) gives:

(24) $$\frac{\partial \hat{y_{i}}}{\partial w}=\sum_{j=1}^{N}\frac{\partial m_{j}\left(x_{i}\right)}{\partial w}\cdot y_{j}+\frac{\partial m^{*}\left(x_{i}\right)}{\partial w}\cdot \frac{\left(\underset{y\in L}{\sup}y+\underset{y\in L}{\inf}y\right)}{2}$$

where the derivative of a single mass in set L is calculated as,

(25) $$\frac{\partial m_{j}\left(x_{i}\right)}{\partial w}=\frac{K\frac{\partial \phi (x_{i},x_{j})}{\partial w}-\frac{\partial K}{\partial w}\cdot \phi \left(x_{i},x_{j}\right)}{K^{2}}\cdot \prod_{k!=i}\left(1-\phi \left(d\left(x,x_{k}\right)\right)\right)\;+\;\frac{\phi (x_{i},x_{j})}{K}\cdot \frac{\partial (\prod_{k!=i}\left(1-\phi \left(d\left(x,x_{k}\right)\right)\right)}{\partial w}$$

With the derivatives of discount function ϕ and the normalization term *K* as,

(26) $$\frac{\partial \phi (x_{i},x_{j})}{\partial w}=-\frac{2}{\gamma }\exp\left(\frac{-d{\left(x_{i},x_{j}\right)}^{2}}{\gamma }\right)\cdot d\left(x_{i},x_{j}\right)\cdot \frac{\partial d(x_{i},x_{j})}{\partial w}$$

(27) $$\frac{\partial K}{\partial w}=\frac{\partial (\prod_{j=1}^{N}\left(1-\phi \left(d\left(x,x_{i}\right)\right)\right))}{\partial w}+\sum_{i=1}^{N}\frac{\partial [\phi \left(d\left(x,x_{i}\right)\right)\prod_{k!=i}\left(1-\phi \left(d\left(x,x_{k}\right)\right)\right)]}{\partial w}$$

The derivative of the product sequence is calculated as,

(28) $$\frac{\partial (\prod_{k!=i}\left(1-\phi \left(d\left(x_{i},x_{k}\right)\right)\right)}{\partial w}=-\sum_{\begin{array}{c} h=1\\ h!=i \end{array}}^{n}\frac{\partial (\phi \left(x_{i},x_{h}\right))}{\partial w}\cdot \prod_{\begin{array}{c} k=1\\ k!=h \end{array}}\left(1-\phi \left(x_{i},x_{k}\right)\right)$$

Finally, the derivative of the mass assigned to the domain can be calculated as,

(29) $$\frac{\partial m^{*}\left(x_{i}\right)}{\partial w}=\frac{K\frac{\partial (\prod_{i=1}^{N}\left(1-\phi \left(d\left(x,x_{i}\right)\right)\right)}{\partial w}-\frac{\partial K}{\partial w}\prod_{i=1}^{N}\left(1-\phi \left(d\left(x,x_{i}\right)\right)\right)}{K^{2}}$$

Then we can apply this calculated gradient, predicting the target variable $y_{i}$ of every example $e_{i}$ in the training set L. When we predict the output value of a sample $e_{i}$, we leave out this sample from set L and use all the other n−1 samples for which the actual output value is known in the set as evidence. We will perform this operation multiple times through all the training set, a single pass through all the training set will be called an epoch. The algorithm will perform a fixed number of epochs. At the end of each epoch the weights will be updated by the calculated gradient multiplied by a learning rate factor α and we will store the weight vector only if a local minimum is found. Finally, the method will return the weight vector $\hat{w}$ which minimizes the loss function *L*, as we show in Algorithm 1.
**Algorithm 1** Weight learning.1: **function**Weight Learning(X,Y,α,NumEpochs)
2:    w←[1,1,⋯,1]
3:    minLoss←∞
4:    $\hat{w}\leftarrow w$
5:    **for**
epoch←(1,NumEpochs)
**do**
6:        $\hat{Y}\leftarrow \left[\;\right]$
7:        **for**$x_{i}\in X$
**do**
8:           $S\leftarrow Remove(x_{i},X)$
▹ Remove $x_{i}$ from training set
9:           $\hat{y_{i}}\leftarrow EVREG\left(x_{i},S,w\right)$
▹ Predict example $e_{i}$ with training set S
10:           $Insert(\hat{y_{i}},\hat{Y})$
▹ Insert $\hat{y_{i}}$ in $\hat{Y}$
11:        **end for**
12:        $loss\leftarrow L(Y,\hat{Y})$
▹ Compute loss
13:        $gradient\leftarrow CalculateGradient(Y,\hat{Y})$
▹ Compute gradient
14:        w←w+α*gradient
▹ Update weights vector
15:        **if**
minLoss>loss**then**
▹ Save best weight vector
16:           minLoss←loss
17:           $\hat{w}\leftarrow w$
18:        **end if**
19:    **end for**
20:    **return**
$\hat{w}$
21: **end function**


The introduction of the weight learning process makes the EVREG model not only better predict the output of a given variable, but also gain the ability to perform feature selection tasks. The main advantage of EVREG is its transparency. This means we can easily track any prediction made by the model; we can get the contribution (mass) of each piece of evidence in the training set L, which makes it intrinsically interpretable. The model can now present a ranking of features according to their importance (weight), i.e., the contribution of a feature to each mass computed, thus helping the end user get a better understanding of her/his data.

## 5. Synthetic Data Experiments

We use synthetic datasets in this section to show the prediction capabilities of WEVREG, testing its prediction performance in various configurations. First we compare its performance with the ones of other well-known regression methods previously used in the health care costs prediction problem. The synthetic datasets that will be used are detailed in Table 3.

These datasets were previously used by Bugata and Drotár [41] to test the performance of WkNN. WkNN is a k-NN based algorithm that, like our method, finds the weight of each feature and then uses a k-NN regressor to make a prediction. WkNN will be one of the methods that will be compared to WEVREG.

The Linear Regression dataset is generated using a random linear regression model, then a gaussian noise with deviation 1 is applied to the output. The Friedman regression problem is a synthetic dataset described in [58], which has only 5 relevant features. The input is uniformly distributed on the interval [0,1]. Again a gaussian noise with 1 standard deviation is applied; the formula is shown in Equation (30).

(30) $$f\left(x\right)=10sin\left(\pi x_{0}x_{1}\right)+20{\left(x_{2}-0.5\right)}^{2}+10x_{3}+5x_{4}+N\left(0,1\right)$$

We used the Mean Absolute Error (MAE) to objectively measure the performance of each method. This error computes the average absolute difference between the predicted cost $\hat{y}$ and the real value *y*, as shown in Equation (31). Where $\hat{y}$ and *y* are vectors of size *n*.

(31) $$MAE\left(\hat{y},y\right)=\frac{1}{n}\sum_{i=1}^{n}\left|\hat{y_{i}}-y_{i}\right|$$

We used three regression methods to compare their performance to the one of WEVREG. The first method was WkNN due to its similarity with WEVREG. Then the two tree-based algorithms to be used were RT and GB. Every method had a unique configuration in each set for simplicity. We used a grid search approach to find the best parameter for each method, except for WkNN where the same configuration as WEVREG was used to demonstrate the difference between both methods. WEVREG used the closest 20 neighbors with a learning rate of 0.1 iterating for 100 times. RT considered all features to create a split and 1 was the minimum number of samples required to be at a leaf node. For GB we used 500 boosting stages and 4 as the minimum number of samples to split a node, with a learning rate of 0.1. The output was scaled to be bound to [0,1]. The performance of these methods is shown in Table 4.

**Figure 4:**
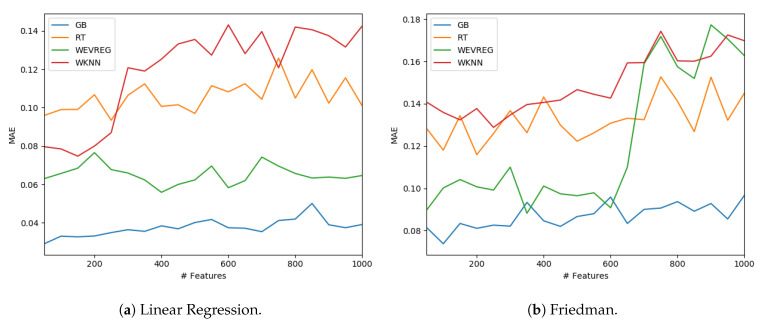
Model performance with different number of features.

GB was the method that obtained the best overall performance as can be observed in Table 4. In particular, WEVREG obtained a good performance resulting in the second best method in most of the sets. WEVREG obtained similar results to WkNN, with slightly better performance of WEVREG. Furthermore, we tested the performance of the methods with a variable number of features. We could observe the performance of each method in the Linear Regression and Friedman dataset using between 50 and 1000 features in Figure 4.

As shown in Figure 4, WEVREG did not decrease its performance with the inclusion of more features in the Linear Regression problem (Figure 4a). This was not the case in the Friedman dataset, where it had an abrupt drop when the 600 features were exceeded, resulting in a performance similar to WkNN. However, WEVREG was able to maintain its performance with the increase of unimportant features for a good portion of the tests, which was more than necessary in the real problem we were trying to solve.

## 6. Experiments and Results

We had access to claims sent by Tsuyama Chuo hospital to the National database and patients’ yearly health checkups, as mentioned in Section 3. We crossed data from both sources to obtain:

- **Demographics**: Patients’ gender and age.
- **Patients’ attributes**: General information about patients such as height, weight, body fat and waist measurement.
- **Health checks**: Results from health check exams a patient had undergone. Japanese workers undergo these exams annually by law. Each exam is indexed by a code, and the result is also included. Some examples are creatinine levels and blood pressure. There were 28 different types of exams, and the date when they were collected was also included.
- **Diagnosis**: Diagnosis for a patient illness registered by date and identified by their ICD-10 codes.
- **Medications**: Detailed information of the dosage and medicine administered, including dates and charges.
- **Costs**: Billing information of each patient’s treatment. Including medicine, procedures and hospitalization costs. The sum of these costs in a year is the value that was predicted.

**Figure 5:**
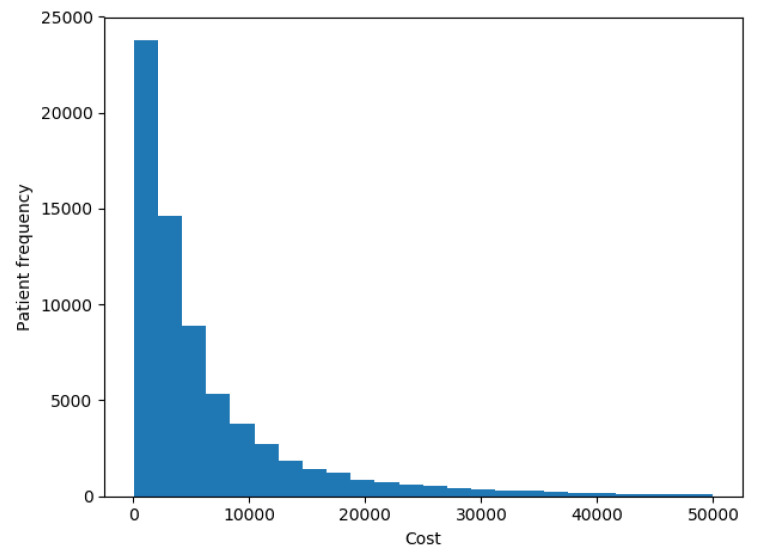
Patients costs distribution.

The goal of our experiments was to test the model presented here and compare it to the results that most successful models reported by the up-to-date literature in terms of accuracy and ability to interpret the results. In this work we tried to predict the costs of each patient in the future year. Figure 5 shows the distribution of patients’ costs. The chart shows that costs had the same distribution as described in [7], with a spike at 0 and a long right-hand tail.

It has been reported [11,12,13] that the use of clinical features (health checks, diagnosis and medications) yields the same performance as using only cost features. This conclusion has been reached without the proper use of an FS algorithm, just by testing the use and omission of clinical data, thus it is not clear whether or not specific clinical data helps to determine a patient’s costs. We will use the clinical features to properly test this hypothesis.

Encoding a patient’s history was done by using all sources available as features. The sources are demographics, health checkups, diagnosis in ICD-10 codification, previous and actual costs.We will only consider chronic conditions for the diagnosis as these can be carried from one year to the next. We will use as chronic conditions the diseases defined by Koller et al. [59] in a study of the impact of multi-morbidity on long-term care dependency. In the study they used 46 chronic conditions based on ICD-10 codes defined by the Central Research Institute of Statutory Ambulatory Health Care in Germany. Table 5 shows a description of the patient’s vector representation with the number of variables of each type in each scenario. As an input vector, we used all dimensions shown in Table 5 except for the actual cost that was used as our target variable.

The evaluation of the performance of our model was done by comparing its results with the methods reported by Sushmita et al. [12], Morid et al. [7] and Duncan et al. [13] for the health cost prediction problem; these works used RT, GB and ANN methods respectively. We also tested a similar regressor algorithm: WkNN.

We used the MAE (Equation (31)) to measure the performance of each method. However, the MAE is useful to compare algorithms in the same set but not to compare results in different datasets, so we also used the Mean Absolute Percentage Error (MAPE), a modified absolute error where the difference between the predicted variable $\hat{y}$ and the real value *y* is divided by value *y*; it is computed as:

(32) $$MAPE\left(\hat{y},y\right)=\frac{1}{n}\sum_{i=1}^{n}\frac{|\hat{y_{i}}-y_{i}|}{y_{i}}$$

where again *n* is the size of vectors $\hat{y}$ and *y*. One disadvantage of this error measure is that it does not support zero values in the output of a dataset. It is expected that most individuals did not incur any cost (healthy individuals). This not the case for our dataset, as we have information of individuals that have concurred to Tsuyama Chuo Hospital for treatment or for a health check. Therefore, we can use MAPE as a performance measure.

We also used another measure, $R^{2}$ which is the Pearson correlation between the predicted and actual health care cost. It represents the proximity of the predicted cost curve to the real cost curve. This value is calculated as:

(33) $$R^{2}\left(\hat{y},y\right)=1-\frac{\sum_{i=1}^{n}{\left(y_{i}-\hat{y_{i}}\right)}^{2}}{\sum_{i=1}^{n}{\left(y_{i}-\bar{m}\right)}^{2}}$$

where $\bar{m}$ is the mean of variable *y* defined as:

(34) $$\bar{m}=\frac{1}{n}\sum_{i=1}^{n}y_{i}$$

**Figure 6:**
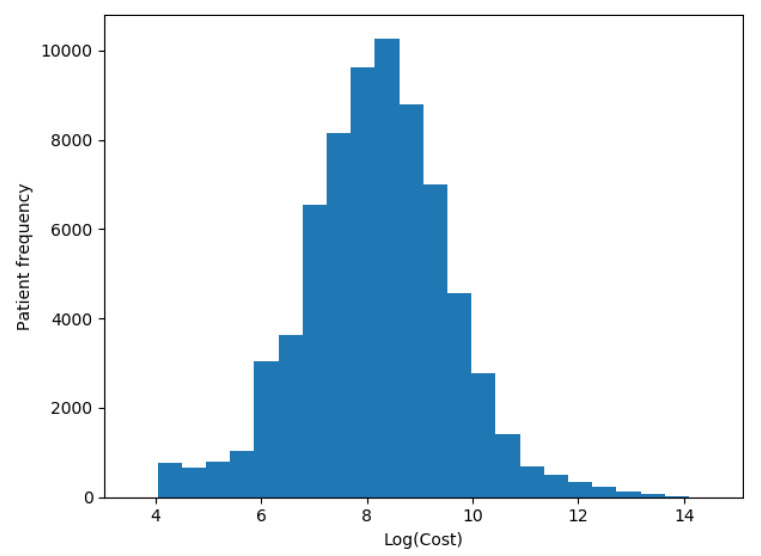
Patients logarithmic costs distribution.

We transformed our target variable (actual costs) to its logarithmic value in each one of the scenarios, as recommended by Diehr et al. [5], so the distribution of patients’ costs in Figure 5 was distributed as shown in Figure 6.

We used a grid search approach for the GB model. We found that the best performance for our dataset was reached when the maximum depth of the individual estimators was 1, the minimum number of samples required to split an internal node was 2 and the number of boosting stages was 125 with a learning rate of 0.1. We tried multiple configurations (number of layers, number of neurons in each layer and activation function) for the ANN; the best configuration had two hidden layers, the first with double the number of inputs and the other with 10, the learning rate was set to 0.01, and with a Rectified Linear Unit (f(x)=max(0,x)) as an activation function. We let it iterate for 30 epochs so it did not overfit. Concerning WkNN configuration, we used 20 neighbors with a Euclidean distance and a RBF kernel. The learning rate was set to 0.01 and was let to iterate for a maximum of 30 epochs. Finally, the WEVREG configuration was the following: we trained the fixed weights for each dimension using gradient descent with a learning rate of 0.1 for 25 epochs, and we used the closest 20 neighbors to improve prediction times, the same quantity used in WkNN.

We performed a 5-fold cross validation procedure to evaluate the performance of each model [60]. The 5-fold cross validation is a statistical procedure where the dataset is randomly divided into five groups, then one of these groups is treated as a validation set and the other four become the training set. This validation is then performed with every group for a total of five times. Finally, the mean performance for each validation group is calculated to obtain the performance of each algorithm. We performed the 5-fold validation five times (resulting in 25 validations) for every tested method. The median of each performance measure is shown in Table 6 with its corresponding standard deviation.

Every method increased its prediction performance through every scenario even though the sets shrank in every step; this could be attributed to the availability of longer history for every patient in the sets. RT was the only method that did not increase its performance drastically with each scenario, obtaining the overall worst performance. The best results across all scenarios were obtained by GB. WEVREG obtained similar results to GB, even obtaining a slightly better $R^{2}$ in the second scenario, but it had a noticeable difference in the last scenario that could be attributed to the smaller size of the set. WkNN obtained worst metrics compared to WEVREG across all scenarios, although it used the same similarity function and similar weight learning. The higher complexity of the masses computation in WEVREG seemed to benefit its prediction power compared to WkNN.

The most important characteristic of WEVREG was its FS capabilities. Weights for each input feature were learned during its training phase. Each feature started with an initial weight of 1, and it was updated in every iteration of the gradient descent. These weights represented the importance of each feature to predict the target variable which in this case was patients’ health care costs. After obtaining the weight of each feature we could select a sub-sample of the features by setting a threshold. We set this threshold to 1, so every weight that increased a little of its value from its starting value would be considered. The top five features on each scenario are shown in Table 7.

As we can observe across each scenario, the most noticeable features were cost features. WEVREG assigned larger weights to the closest previous costs. These results are in line with previous works [11,12,13], where it was reported that cost features alone are a good indicator for future health expenses.

Patient’s age seemed to be a small differentiable feature, that had a small effect in the search of similar patients. Diagnosis features and health checkups features did not have a noticeable effect for health care costs prediction. In the case of the last scenario, even though it was overshadowed by cost features, diabetes mellitus was a meaningful diagnosis to reach a correct cost prediction. This could be related to the fact that patients with this diagnosis incurred high expenses attributed to inpatient care [61,62]. An important feature across all scenarios was the diagnosis of dementia in the previous year; it seemed to be a small factor to group patient with similar costs in cases where it was present.

We used the weights learned by the model to filter out unnecessary features. The selected features were the ones that had been assigned a weight larger than 1. Table 8 shows the number of selected features by scenario.

We tested once more the performance of the methods with significantly smaller sets in each scenario. The results can be seen on Table 9.

Every model, except GB, increased its performance in all scenarios, as observed in Table 9. In particular, WkNN and RT had a significant improvement in its MAE and $R^{2}$ scores. ANN and WEVREG had a high improvement in the last scenario, which could be attributed to the large number of unnecessary features in the first place. The initial features seemed to induce too much noise for the models to make precise predictions. On the other hand, GB did not seem to be benefited as the other models with the filtered features since it obtained similar performances, showing the GB resilience in the presence of noise in its features compared to the other tested models. These results show the capability of WEVREG to complete FS tasks, allowing us to decrease the number of features considerably, speeding up training times and model performance. They also show that the most important features for health care costs prediction were cost features; other features such as demographics and some diagnosis such us diabetes mellitus helped to determine a patient’s costs but were not as indicative as previous costs.

## 7. Discussion and Conclusions

We presented a new regression method with the ability to do FS tasks; it has comparable prediction performance as state of the art regression methods. It can easily show the most relevant features for health care cost prediction; in particular, we obtained that cost features for our dataset are the most relevant to determine a patient’s future health care costs.

Since WEVREG uses a k-NN approach we can easily keep track of how a prediction is made; this is a desirable ability in the health care domain. We compared its results with the predictions made by four models; two of them (GB and ANN) are the best ones from the eleven models analyzed and reported by Morid et al. [7]. When comparing results we can conclude that our method obtains comparable performances to these methods, proving that it is possible to create and use more transparent models for a regression problem like health care cost prediction, challenging the common belief that complex and black-box like methods are always the solution with the best performance for every problem being presented.

We improved Evidential regression presented by Petit-Renaud and Denœux [10] to be used in the prediction of health care costs. Our results were obtained using data of electronic health records from Tsuyama Chuo Hospital. The results showed that our Weighted Evidential Regression method obtained $R^{2}=0.44$ in the best scenario, which shows that a transparent and interpretable method, could perform as current state of the art supervised learning algorithms such as ANN ($R^{2}=0.44$) and GB ($R^{2}=0.49$).

Even though results are similar or better than previous works, we believe our results are still improvable. One of the approaches to improve performance is classifying patients in cost buckets as recommended by various studies [7,11,13]; this strategy results in better performance but escapes the goal of this work, so we can apply this classification process in future work to obtain a patient’s risk class as first step to try to improve the performance of WEVREG. Another approach could be the use of a nearness to death feature as it has been found that costs rise sharply with it [26,27,28]. It is impossible to know a patient’s near death status with our current data. We could include census data to create the new feature, obtaining nearness to death by age group taking into account the change in life expectancy depending on patients’ age [63]. We can also try to solve the prediction of health care cost using deep learning methods, but this purpose may become feasible with the availability of a larger dataset. Finally, we also plan to apply this model to other regression problems in the health care domain, such as predicting the hospital length of stay and predicting the days of readmission based on each patient’s diagnosis and history, which are two classic prediction problems in this domain.

## Figures and Tables

**Table 1:** Insurance claims.

Header	Name	Description
IR	Medical institution	Details of the medical institution.
RE	Insured details	Patient details with dates and demographics.
HO	Insurer details	Patient insurer information.
KO	Public expenses	Patient public expense information.
KH	Special information	Patient especial information (free text).
SY	Diagnosis	Patient diagnosis in MHLW coding.
SI	Procedure	Details for a patient treatment.
IY	Medications	Details for the medicines given.
TO	Specific equipment	Specific equipment details used in a patient.
CO	Comment	Comments for diagnoses or symptoms (free text).
SJ	Symptoms	Patients symptoms.

**Table 2:** Statistics of patients’ records in each scenario.

Statistics	Scenario 1	Scenario 2	Scenario 3
Total number of patients	71,001	33,646	8810
Mean costs	11,030	11,536	12,420
Mean age	54.00	58.00	63.00
% Male	48.81	48.54	48.56
% Female	51.19	51.46	51.44

**Table 3:** Synthetic regression datasets.

Name	Samples	Relevant Features	Total Features
Linear Regression	200	5	500
Friedman	200	5	500
Linear Regression 5k	5000	5	500
Friedman 5k	5000	5	500

**Table 4:** MAE on synthetic datasets (the lower the better).

Name	RT	WkNN	WEVREG	GB
Linear Regression	0.11±0.03	0.08±0.02	0.08±0.02	0.03±0.01
Friedman	0.12±0.01	0.14±0.01	0.10±0.01	0.08±0.01
Linear Regression 5k	0.06±0.01	0.02±0.00	0.03±0.00	0.01±0.00
Friedman 5k	0.09±0.01	0.08±0.01	0.07±0.01	0.05±0.01

**Table 5:** Number of variables by type in patient encoding.

Description	Scenario 1	Scenario 2	Scenario 3
Demographics	2	2	2
Health checkups	27	54	135
Chronic diseases	46	92	230
Medication info	2	4	10
Previous costs	1	2	5
Actual cost	1	1	1

**Table 6:** Models performance with all features (the lower is the better for Mean Absolute Error (MAE) and Mean Absolute Percentage Error (MAPE); the higher the better for $R^{2}$).

Model	Scenario 1			Scenario 2			Scenario 3		
	**MAE**	**MAPE**	$R^{2}$	**MAE**	**MAPE**	$R^{2}$	**MAE**	**MAPE**	$R^{2}$
RT	0.97±0.01	0.13±0.00	0.16±0.01	0.94±0.01	0.12±0.00	0.18±0.02	0.91±0.02	0.11±0.00	0.17±0.02
WkNN	0.95±0.01	0.12±0.00	0.21±0.01	0.91±0.00	0.12±0.00	0.25±0.00	0.82±0.01	0.10±0.00	0.34±0.01
ANN	0.92±0.01	0.13±0.00	0.22±0.01	0.85±0.01	0.11±0.00	0.30±0.01	0.79±0.03	0.10±0.00	0.35±0.04
WEVREG	0.92±0.02	0.12±0.00	0.23±0.02	0.84±0.00	**0.11** ±0.00	0.33±0.00	0.75±0.02	0.09±0.00	0.41±0.02
GB	0.89±0.02	0.12±0.00	0.26±0.02	0.84±0.01	0.11±0.00	0.32±0.01	0.67±0.02	0.09±0.00	0.49±0.02

**Table 7:** Top 5 features for each scenario.

Scenario 1		Scenario 2		Scenario 3	
**Feature**	**Weight**	**Feature**	**Weight**	**Feature**	**Weight**
*cost_1*	65.06	*cost_1*	19.67	*cost_5*	19.66
*age*	6.19	*cost_2*	16.47	*cost_4*	19.66
*gender*	1.09	*age*	3.09	*cost_3*	19.66
*dementia_1*	1.03	*urinary_incontinence_1*	1.70	*cost_2*	19.66
*parkinsons_disease_1*	1.03	*dementia_2*	1.59	*diabetes_mellitus_3*	2.43

**Table 8:** Number of features selected by scenario.

Scenario	Total Features	Selected Features
1	76	5
2	154	10
3	382	33

**Table 9:** Model performance with selected features (for MAE and MAPE lower is better, for $R^{2}$ higher is better).

Model	Scenario 1			Scenario 2			Scenario 3		
	**MAE**	**MAPE**	$R^{2}$	**MAE**	**MAPE**	$R^{2}$	**MAE**	**MAPE**	$R^{2}$
RT	0.92±0.02	0.13±0.00	0.22±0.03	0.87±0.03	0.12±0.00	0.27±0.03	0.81±0.02	0.10±0.00	0.32±0.02
WkNN	0.91±0.01	0.12±0.00	0.23±0.01	0.82±0.01	0.11±0.00	0.34±0.01	0.76±0.02	0.10±0.00	0.41±0.02
ANN	0.92±0.01	0.13±0.00	0.22±0.01	0.83±0.02	0.11±0.00	0.32±0.02	0.72±0.03	0.09±0.00	0.44±0.03
WEVREG	0.91±0.01	0.12±0.00	0.23±0.01	0.82±0.01	0.11±0.00	0.34±0.01	0.72±0.02	0.09±0.00	0.44±0.02
GB	0.91±0.01	0.12±0.00	0.24±0.01	0.82±0.01	0.11±0.00	0.33±0.01	0.68±0.02	0.08±0.00	0.48±0.02
